# GC–MS based metabolomic profiling of *Aporosa cardiosperma* (Gaertn.) Merr. leaf extracts and evaluating its therapeutic potential

**DOI:** 10.1038/s41598-024-66491-2

**Published:** 2024-07-11

**Authors:** Ubais Abdul, Dinesh Babu Manikandan, Manikandan Arumugam, Suliman Yousef Alomar, Salim Manoharadas, Thirumurugan Ramasamy

**Affiliations:** 1 Laboratory of Aquabiotics/Nanoscience, Department of Animal Science, School of Life Sciences, Bharathidasan University, Tiruchirappalli, Tamil Nadu 620 024 India; 2 Department of Zoology, College of Science, King Saud University, Riyadh, 11451 Kingdom of Saudi Arabia; 3 Department of Botany and Microbiology, College of Science, King Saud University, Riyadh, 11451 Kingdom of Saudi Arabia; 4 Present Address: Central University of Tamil Nadu, Thiruvarur, Tamil Nadu 610 005 India

**Keywords:** Biotechnology, Microbiology, Plant sciences

## Abstract

*Aporosa cardiosperma* is a plant species majorly found in the Indian Western Ghats that belongs to the *phyllanthaceae* family with ethnobotanical importance. Using a Fourier Transform-Infrared Spectrometer (FT-IR) and Gas Chromatography-Mass Spectrometry (GC–MS) for evaluating leaf extracts of *A. cardiosperma*, significant functional groups and metabolite constituents were determined, and its total flavonoid, phenol, and tannin content were quantified. Further, its antibacterial efficacy was investigated against microorganisms that cause fish and human disease and are resistant to common antibiotics, including *Staphylococcus aureus, Bacillus subtilis, Mycobacterium tuberculosis, Klebsiella pneumoniae*, *Aeromonas hydrophila*, and *Pseudomonas aeruginosa*. Regarding the outcomes of GC–MS analysis, the primary metabolites in the *A. cardiosperma* leaf extracts were heneicosane (57.06%), silane (13.60%), 1-heptadecene (10.09%), 3-hexadecene (9.99%), and pentadecane (9.54%). In comparison to other solvents, methanolic extract of *A. cardiosperma* leaves had increased phenolic, flavonoid, and tannin content; these findings are consistent with in vitro antioxidant potential and obtained that the methanolic extract (100 µg/mL) exhibited the higher percentage of inhibition in DPPH (82.35%), FRAP (86.20%), metal chelating (72.32%), and ABTS (86.06%) antioxidant assays respectively. Similar findings were found regarding the antibacterial efficacy against pathogenic bacteria. Comparatively, to other extracts, methanolic extracts showed more significant antibacterial activity at a lower minimum inhibitory concentration (MIC) value (250 µg/mL), whilst ethyl acetate and hexane solvent extracts of *A. cardiosperma* leaves had higher MIC values 500 µg/mL and 1000 µg/mL respectively. The antimicrobial potential was validated by investigating bacterial growth through the extracts acquired MICs and sub-MICs range. Bacterial growth was completely inhibited at the determined MIC range. In conclusion, *A. cardiosperma* leaf extract's phytochemical fingerprint has been determined, and its potent antibacterial and antioxidant activities were discovered. These findings of the current study will pave the way for developing herbal treatments from *A. cardiosperma* for various fish and human diseases.

## Introduction

Medications with various chemical compositions can cause adverse effects, from mild discomfort to severe outcomes. The Food and Drug Administration (FDA) monitors and takes action to ensure safety and report adverse effects^1^. Herbal medicines are eco-friendly, safe, and offer diverse benefits: antioxidants, antibacterial, anti-inflammatory, and anticancer properties^2^, that widely utilized in folk heals to treat a range of ailments, such as inflammatory, neoplastic, diabetic, hypertensive, and cardiovascular ailments^3^. Medicinal plants provide compounds for innovative pharmaceuticals. The *phyllanthaceae* family, with more than 1000 species worldwide, holds significant potential as an antioxidant, anthelmintic, antibacterial, anti-diarrheal, anti-tumor, anti-inflammation, and insecticidal^4^. *A. cardiosperma,* abundantly found in the Western Ghats of South India, provides carbohydrates and mineral-rich fruit. Its mucilage, and tannin-rich cells protect various pests^5^. *Aporosa* genus including *A*. *lindleyana*^6^, *A. wallichii*^7^, and *A. octandra*^8^ are used in various medicinal applications. Plant metabolites are used in aquaculture for nutrition, health, water quality, disease control, and environmental sustainability benefits. Aquaculture is the fastest-growing sector in food production worldwide, supplying 50% of global fish consumption^9^. Disease emergence in freshwater aquaculture is a significant concern by pathogens like *S. aureus*, *B. subtilis*, *M. tuberculosis*, *K. pneumoniae*, *P. aeruginosa*, and *A. hydrophila* can cause contagious diseases in fish and humans^10^, when exposed to antibiotics, these pathogens develop resistance, posing risks to humans and the environment^11^. To overcome this limitation, plant-based drugs, which have been used historically as immune boosters, offer aquaculture-safe, natural alternatives to antibiotics^12^. Interest in these plants has surged globally due to their simplicity, affordability, and minimal adverse effects on ecosystems and animal life^13^. Over 30,000 antimicrobial compounds from plants and 1340 species with antibacterial properties have been identified^14^. Between 14 and 28% of diverse plant species are predicted to possess therapeutic qualities, with 74% of their bioactive compounds explored for medical applications^15^. Antibacterial resistance stems from widespread, inappropriate antibiotic use, rendering many drugs ineffective^16^. The World Health Organization (WHO) observed a significant obstacle to medical progress, emphasizing the need for alternative antibiotics to curb resistance^17^. Researchers are urged to isolate and identify novel bioactive compounds from plants to combat microbial resistance in aquaculture. Still, many medicinal plants possess antibacterial properties are undiscovered; efforts focus on developing rapid, advanced, and efficient treatments^13^. With this evidence, this study investigates the medicinal plant *A. cardiosperma* for its antibacterial and antioxidant properties by evaluating the phytochemical composition and metabolites to uncover its potential therapeutic applications.

## Materials and methods

### Materials required

Solvents used in the experiments are methanol (99%, CDH, New Delhi, India), ethyl acetate (99.5%, Loba chemie, Mumbai, India), and hexane (85%, Molychem, Mumbai, India). Chemicals and reagents required are sulfuric acid (98%, Merck, Mumbai, India), sodium hydroxide (97%, Rankem, Maharashtra, India), chloroform (99%, Loba chemie, Mumbai, India), dragendorff’s reagent (Loba chemie, Mumbai, India), benedict reagent (qualitative, Loba chemie, Mumbai, India), copper sulphate (99%. Himedia, Mumbai, India), aluminum chloride (98%, Merck, Mumbai, India), gallic acid (98%, SRL, Maharashtra, India), folin ciocalteu reagent (Himedia, Mumbai, India), sodium carbonate (99.5%, Merck, Mumbai, India), sodium nitrate (98%, Merck, Mumbai, India), tannic acid (Rankem, Maharashtra, India), quercetin (99%, SRL, Maharashtra, India), DPPH (95%, SRL, Maharashtra, India), ascorbic acid (99.7%, SRL, Maharashtra, India), iron-ferrozine (Biosystem, Tamil Nadu, India), trichloroacetic acid (98%, Fisher scientific, Mumbai, India), potassium ferricyanide (99%, Fisher scientific, Mumbai, India), ferric chloride (98%, Loba chemie, Mumbai, India), ABTS (98%, Himedia, Mumbai, India), potassium persulphate (Himedia, Mumbai, India), ethanol absolute (99.9%, CSS, Jiangsu, China), nutrient agar (Himedia, Mumbai, India), nutrient broth (Himedia, Mumbai, India), streptomycin (95%, Himedia, Mumbai, India), DMSO (99.05%, Himedia, Mumbai, India), resazurin dye (Himedia, Mumbai, India).

### Plant collection

The leaves of *A. cardiosperma* were collected from the Achankovil, Kollam district, Kerala, India. Sample plant situated within geographic coordinates 76° 58′ 08.3″ E–W latitude and 9° 03′36.4″ N–S longitudes. Field studies and experimental research on the methods of collecting medicinal plant material were conducted under the guidelines and regulations provided by the National Medicinal Plants Board, Department of AYUSH, Ministry of Health and Family Welfare, Government of India (2009). *A. cardiosperma* grows in nature abundantly and does not require permissions. Taxonomist Dr. M.U. Sharief, Scientist ‘F’ and Head of Office, Ministry of Environment, Forest and Climate Change, Botanical Survey of India (BSI), Southern Regional Centre, Coimbatore, Tamil Nadu, India, has verified the species and deposited in the institutional Herbarium (Herbarium deposition No: BSI/SRC/5/23/2023/Tech-617).

### Leaf extracts of *A. cardiosperma*

Fresh plant leaves were washed by rinsing them with water, allowed to dry in the shade for two weeks, and then milled into the fine powder. The dry powdered leaf was extracted using various solvents based on polarity, including polar (methanol), mid-polar (ethyl acetate), and non-polar (hexane), for two days of shaking in the shaker. Then, the solvents are filtered and concentrated by evaporating solvents using a hot-air oven for 3 days at 40 °C temperature^18^. The resultant extracts were stored at 4 °C for further studies.

### Phytochemical screening of *A. cardiosperma* leaf extracts

The dried *A. cardiosperma* leaves powder, weighing 10 g, was mixed with 100 mL of the aformentioned solvents and filtered for the phytochemical analysis. Such as tannins, phlobatannins, saponins, flavonoids, phenolic flavonoids, terpenoids, steroids, phytosteroids, phenol, alkaloids, carbohydrates, and protein are present in various extracts of *A. cardiosperma* leaves investigated using Arumugam et al.^18,19^ methods.

#### Total phenol content (TPC)

Intially, 0.5 mL of *A. cardiosperma* leaf prepared extracts were taken and diluted with 8 mL of distilled H_2_O. Then, 0.5 mL of Folin Ciocalteu reagent (1 N) was added and kept at 40 °C for 10 min. Further, 1 mL of sodium carbonate (20%) was added and kept in the dark for 1 h. The absorbance was read at 765 nm using a UV visible spectrophotometer Synergy HT Multimode Reader^19^ (Biotek, Winooski, USA). The same procedure was repeated for all standard gallic acid solutions, and the standard calibration curves were obtained. The sample concentration was calculated as gallic acid equivalent (GAE)/50 g.

#### Total tannin content (TTC)

Folin-Ciocalteu's phenol reagent was used to quantify the total tannin content of *A. cardiosperma* leaf extracts by the Arumugam et al.^19^ procedure. In 100 μL of the leaf extract, 8.3 mL of distilled H_2_O and 0.5 mL of Folin-Ciocalteu phenol reagent were added in a test tube and incubated for 5 min at 37 °C. Then, 1 mL of 35% Na_2_CO_3_ solution was mixed in the test tube. The mixture was shaken gently and undisturbed at 25 ± 2 °C for 30 min. The absorbance was measured at 725 nm, and distilled H_2_O was used as the blank. Tannic acid served as the standard reference, and the total tannin content of the extracts was reported in mg of tannic acid equivalent (TAE)/50 g.

#### Total flavonoid content (TFC)

Intially, 0.5 mL of the prepared leaf extracts of *A. cardiosperma* were diluted with 3.5 mL of distilled H_2_O, followed by the addition of 0.3 mL of 5% sodium nitrate to the tube. After 5 min, the tube was added with 0.3 mL of 10% aluminum chloride. The mixture dissolved with 2 mL of sodium hydroxide (1 M) in the 6th min. The contents of the reaction mixture were thoroughly combined and promptly diluted with 2.4 mL of distilled H_2_O. The mixtures were read at 414 nm with the comparison to a blank. Quercetin was the standard compound used to quantify total flavonoids and expressed as mg of quercetin equivalent (QE)/50 g^20^.

### FT-IR analysis

The functional groups of the *A. cardiosperma* leaf extracts were investigated utilizing the KBr pellet method with spectra of 4000–400 cm^−1^ through an FT-IR spectrophotometer (Perkin Elmer, USA)^19^.

### GC–MS metabolite profiling

#### Preparation of *A. cardiosperma* leaf extracts

The proportion of *A. cardiosperma* leaves was shade dried out, and then 10 g of the powdered leaf was mixed with 100 mL of solvents (methanol, ethyl acetate, and hexane), respectively. Using a Soxhlet apparatus, the solvents were evaporated under reduced pressure at 55–60 °C and dried in a vacuum. The residue was filtered and concentrated into a dry mass by a rotary vacuum evaporator. Then, extracts were filtered and loaded in the glass vial using a sterile syringe filter with a 0.22 µm pore size^18^.

#### GC–MS analysis of *A. cardiosperma* leaf extracts

Gas chromatography–mass spectrometry analysis (GC–MS—QP-2010 Plus, Shimadzu, Tokyo, Japan) was performed on the solvents containing *A. cardiosperma* leaf extracts using the thermal desorption (TD) system 20. The GC–MS system operated under specified conditions, which included an RTX-5MS capillary column (30 m × 0.25 mm with a film thickness of 0.25 mm), 1.5 mL/min of helium gas (serving as a carrier), 250 °C for the gun, 290 °C for the detector, and 60–180 °C for the column at a rate of 5 °C/min, followed by 180–280 °C at 10 °C/min (10 min), 0.5 scan/s of *m/z* 40 and 350, and a split ratio of 1:200. One microliter of the prepared sample was used as the injector volume. To avoid the noise peaks, the solvents were passed through the column before the sample injection^19^. Along with retention indices, the results were cross-checked against previously published literatures using the Wiley 08 spectral Library Searching Programme (http://www.sisweb.com/software/ms/wiley-search.html) and NIST mass 17 spectrum libraries.

###  In-vitro antioxidant activity of *A. cardiosperma* leaf extracts

In the present study, the absorbance of the control solution [substrate (DPPH, FRAP, ABTS, Metal chelating) in solvent without leaf extracts of *A. cardiosperma*] was adjusted to zero to represent the absorbance of the blank solution during the antioxidant tests. Using the formula (Eq. 1), which determines the percentage of inhibition, whereas A_test_ represents the absorbance of the reaction mixture containing *A. cardiosperma* leaf extracts/standard, A_cont_ represents the absorbance of the control solution^21^. Reduced absorbance was denoted by the significance of higher free-radical scavenging capacity.

1 $${\text{Percentage of inhibition }} = \, \left[ {\left( {{A_{{\text{cont}}}} - {A_{{\text{test}}}}} \right)/{A_{{\text{cont}}}}} \right] \, \times \, 100$$

### DPPH radical-scavenging assay

This experiment assessed the *A. cardiosperma* leaf extracts capacity to scavenge free radicals, following the detailed protocol provided by Brand-Williams et al.^22^. The procedure involved mixing 1.0 mL of 0.1 mM DPPH in methanol with 1.0 mL of various concentrations of *A. cardiosperma* leaf extracts (20–100 µg/mL), shaking thoroughly, and then the mixture was kept undisturbed in the dark for 30 min at room temperature. The absorbance of the mixture was measured at 517 nm. Ascorbic acid was used as the reference standard. The percentage of inhibition for DPPH radical scavenging activity was calculated using the Eq. (1).

### FRAP assay

The reducing power of *A. cardiosperma* leaf extracts was ascertained using the El Jemli et al^2^ approach. This experiment involved mixing different concentrations of *A. cardiosperma* leaf extracts (20–100 µg/mL) with 2.5 mL of phosphate buffer (200 mM, pH 6.6) and 2.5 mL of 1% potassium ferricyanide. The mixture was then incubated at 50 ºC for 20 min, followed by rapid cooling. After adding 2.5 mL of 10% TCA (trichloroacetic acid) to the mixture, centrifuging it for 10 min at 3000 rpm. The top layer was removed and added with the same amount of distilled H_2_O. Finally, the prepared solution was mixed with 1 mL of 1% ferric chloride (FeCl_3_) and read at 700 nm. An increase in ferric reducing power was inferred from the reaction mixture's increased absorbance. Ascorbic acid, the standard was prepared using the same procedure. The percentage of reducing power was calculated using Eq. (1).

### Metal-chelating assay

They were investigating the metal-chelating activity of *A. cardiosperma* leaf extracts and standard antioxidants using the Dinis et al.^23^ approach. The different extract concentrations (20–100 µg/mL) were added to 0.05 mL of 2 mM FeCl_2_; the mixture was left to incubate at room temperature for 5 min. The reaction began with the addition of ferrozine (5 mM; 0.3 mL). The mixture was shaken vigorously after an incubation of 10 min at room temperature. Ascorbic acid served as a positive control. The absorbance was read at 562 nm. Equation (1) was used to calculate the inhibition percentage of the formation of the Fe^2+^-ferrozine complex.

### ABTS radical scavenging assay

The antioxidant qualities of various *A. cardiosperma* leaf extracts were assessed using the ABTS radical cation decolorization test^18^. In deionized water, 88 μL of 140 mM potassium persulphate solution was used to dissolve 7 mM ABTS. The mixture was undisturbed for 14 h at 37 °C in the dark. The ABTS mixture was diluted in ethanol (1:89 v/v) to achieve an absorbance. Then, 200 μL of freshly prepared ABTS^+^ solution was mixed with 100 μL of solvent extracts at different concentrations (20, 40, 60, 80, and 100 μg/mL) and incubated for 10 min. The absorbance of the solution mixtures was read at 734 nm. The standard employed was ascorbic acid. Equation (1) was used to calculate the ABTS radical scavenging activity.

### In-vitro antibacterial efficacy of *A. cardiosperma* leaf extracts

#### Agar well diffusion method

*In-vitro* antibacterial activity of *A. cardiosperma* extracts was evaluated using the agar well diffusion method^24^. The Microbial Type Culture Collection and Gene Bank (MTCC), located in Chandigarh, India, provided the pure culture of the bacterial strains. These bacterial stains, including gram positive [*S. aureus* (MTCC 96)*, B. subtilis* (MTCC 736)*, and M. tuberculosis* (clinical isolate)] and gram negative [*A. hydrophila* (MTCC 646)*, K. pneumoniae (*MTCC 109), *and P. aeruginosa* (MTCC 2488)] were cotton-swabbed on the freshly prepared nutrient agar plates. A cork borer was used for the gel puncture to make 6 mm diameter wells on nutrient agar plates. Various *A. cardiosperma* solvent extract concentrations (250, 500, 750, and 1000 μg/mL) were added to the corresponding wells along with positive control [streptomycin (25 μg/mL)]. Then, the petri plates were incubated for 24 h at 37 °C, then the zone of inhibition (mm) was measured. The experiments were carried out in triplicates.

### Minimum inhibitory concentration

To estimate the MIC for each *A. cardiosperma* leaf extracts, a sterile 96-well microtiter plate containing resazurin as a cell growth indicator was utilized^18^. After sterilizing the laminar air flow chamber, a 96-well microtiter plate, 100 μL of freshly prepared nutrient broth, and 100 μL of various extracts (10 mg/mL). DMSO [10% (v/v)] was added as control without *A. cardiosperm*a leaf extracts to the third row of the plate. Then, 10 μL of resazurin dye and 10 μL of inoculum of bacterial strains (5 × 10^6^ CFU/mL) was added to each well. After that, the microtiter plate was carefully wrapped with an aluminum foil to avoid the dehydration of the bacterial culture and it was kept at 37 °C for 24 h. Visual inspection of the wells revealed that color shifts from purple to pink (positive) or colorlessness were considered negative. All the experiments were carried out in triplicates.

### Bacterial growth kinetics

Using a modified Arumugam et al.^19^ protocol, the growth of *S. aureus, B. subtilis, M. tuberculosis, A. hydrophila, K. pneumoniae, and P. aeruginosa* were investigated using the determined MIC and sub-MIC (1/2 MIC) of *A. cardiosperma* leaf extracts. Extracts were added based on the obtained MIC and sub-MIC values (in triplicates) after microbial cultures were added into tubes to achieve a final inoculum of 1.6 × 10^4^ CFU mL^−1^, and the culture inoculated plates were maintained at 37 °C. Using a UV–visible spectrophotometer (Synergy HT Multimode Reader, Biotek equipment, Winooski, VT, USA), the bacterial growth was measured spectrophotometrically for 24 h at 2-h intervals by measuring the optical density at 600 nm. The streptomycin (25 µg/mL) was used as the positive control, and the well-untreated bacterial cultures acted as the negative control.

### Data analysis

All the experiments were carried out in triplicates, and the results were expressed in the mean ± standard error of the mean (SEM). Applying one-way ANOVA with Dunnett's multiple comparisons test^25^ and two-way ANOVA with Tukey's multiple comparisons test^26^ on the data was analyzed using GraphPad Prism version 8 (GraphPad Software, Inc., San Diego, CA). The data were presented as descriptive statistics through tables and graphs. *, **, ***, and **** indicate P-values of respectively ≤ 0.05, ≤ 0.01, ≤ 0.001, and ≤ 0.0001 and “ns” indicates no significant.

## Results

### Phytochemical screening of *A. cardiosperma* leaf extracts 

**Figure 1:**
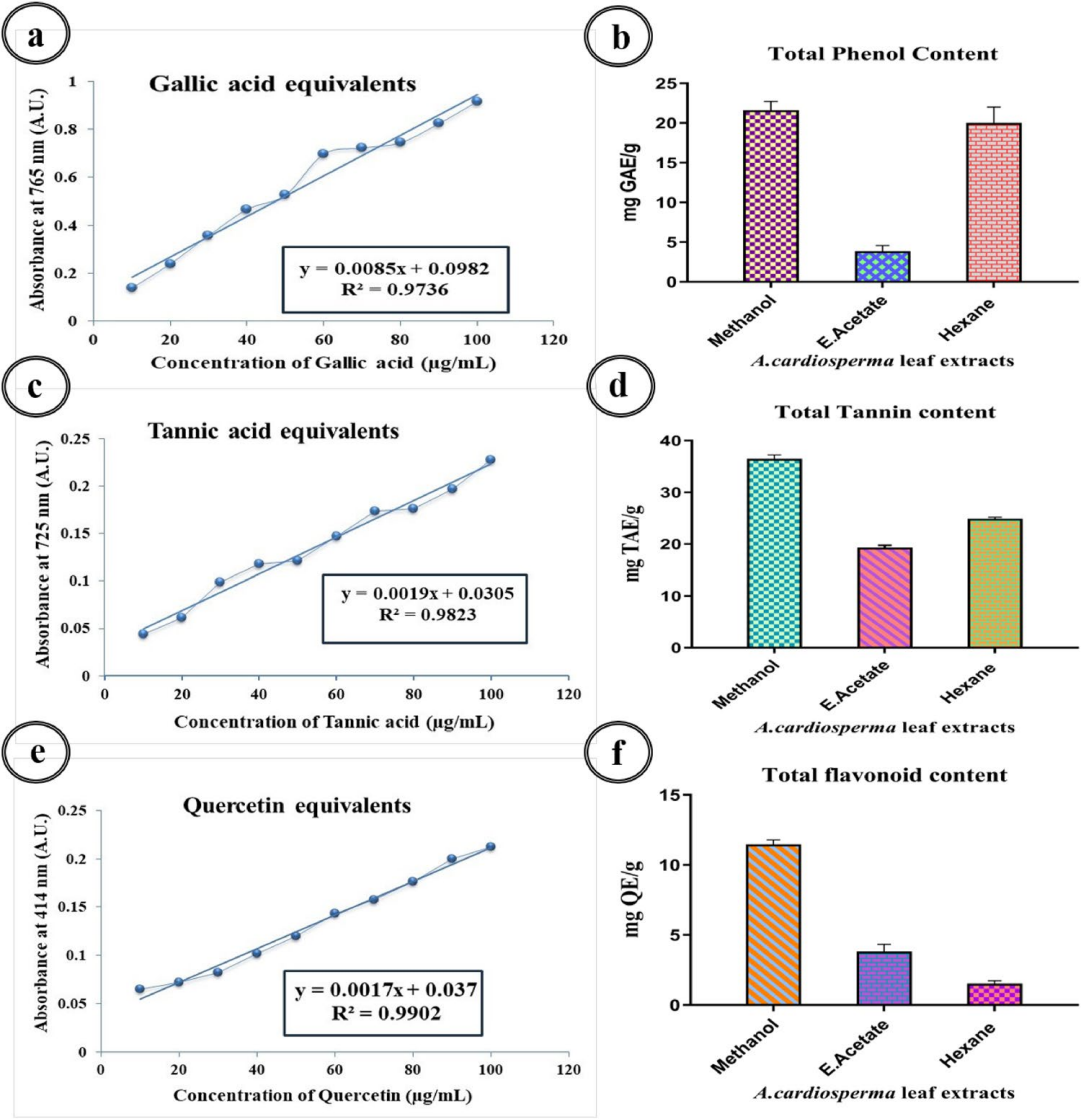
Standard graph of Gallic acid equivalents (**a**), Quantitative analysis of the total phenolic content (**b**), Standard graph of Tannic acid equivalents (**c**) and Quantitative analysis of the total tannin content (**d**), Standard graph of Quercetin equivalents (**e**), Quantitative analysis of the total flavonoid content (**f**) present in the various extracts of *A. cardiosperma* leaf.

The phytochemical analysis of *A. cardiosperma* leaf used polar solvents (aqueous, methanol and ethanol) extracts revealed tannins, phlobatannins, saponins, flavonoids, phenolic flavonoids, terpenoids, steroids, phytosteroids, phenols, alkaloids, carbohydrate, and proteins. In contrast, the mid-polar solvents (ethyl acetate, acetone) extract revealed tannins, phlobatannins, saponins, flavonoids, phenolic flavonoids, terpenoids, steroids, phytosteroids, phenols, alkaloids, carbohydrates, and proteins. The non-polar solvents (petroleum ether and hexane) extract contained tannins, phlobatannins, saponins, flavonoids, phenol, steroids, and carbohydrates (Table 1). The total phenolic quantity was found to be higher in methanolic extract (21.61 ± 1.111 mg/GAE g extract), followed by hexane and ethyl acetate extracts of *A. cardiosperma* leaf (20.02 ± 1.997 mg/GAE g and 3.86 ± 0.703 mg/GAE g) (Fig. 1). Total tannin content of methanolic extract shows higher quantity (36.45 ± 0.793 mg/TAE g extract) compared to the hexane (24.89 ± 0.249 mg/TAE g) and ethyl acetate (19.28 ± 0.471 mg/TAE g extract) respectively. The total flavonoid content of the methanolic is higher (11.47 ± 0.309 mg/QE g), followed by ethyl acetate (3.82 ± 0.519 mg/QE g) and hexane (1.53 ± 0.191 mg/QE g).

**Table 1:** Preliminary phytochemical screening of various extracts of *A. cardiosperma* leaf.

Plant constituents	Aqueous extract	Methanol extract	Ethanol extract	Acetone extract	Ethyl acetate extract	Petroleum ether extract	Hexane extract
Tannin	+ + +	+ +	+	−	+	−	+
Phlobatannins	+ + +	+ + +	+ +	+	+ +	+	+
Saponin	+ + +	+ +	+	+ +	+	+	+
Flavonoids	+ + +	+ + +	+ +	+ +	+	+	+
Phenolic flavonoids	+ + +	+	+	−	−	−	−
Terpenoids	+ + +	+ + +	+ +	+ +	−	−	−
Steroids	+ +	+	+ + +	+ +	−	−	+
Phyto steroids	+ +	+ +	+ +	+ +	−	−	−
Phenol	+ + +	+ + +	+ + +	+ +	+	−	+ +
Alkaloids	+ + +	+ + +	+	+	+	−	−
Carbohydrate	+	+	+	+	+	+	+
Protein	+	+	+	+	+	−	−

(+++ : Highly ++ : Moderate + : Present −: Absent).

### Characterization of *A. cardiosperma* leaf extracts using FT-IR spectroscopy

**Figure 2:**
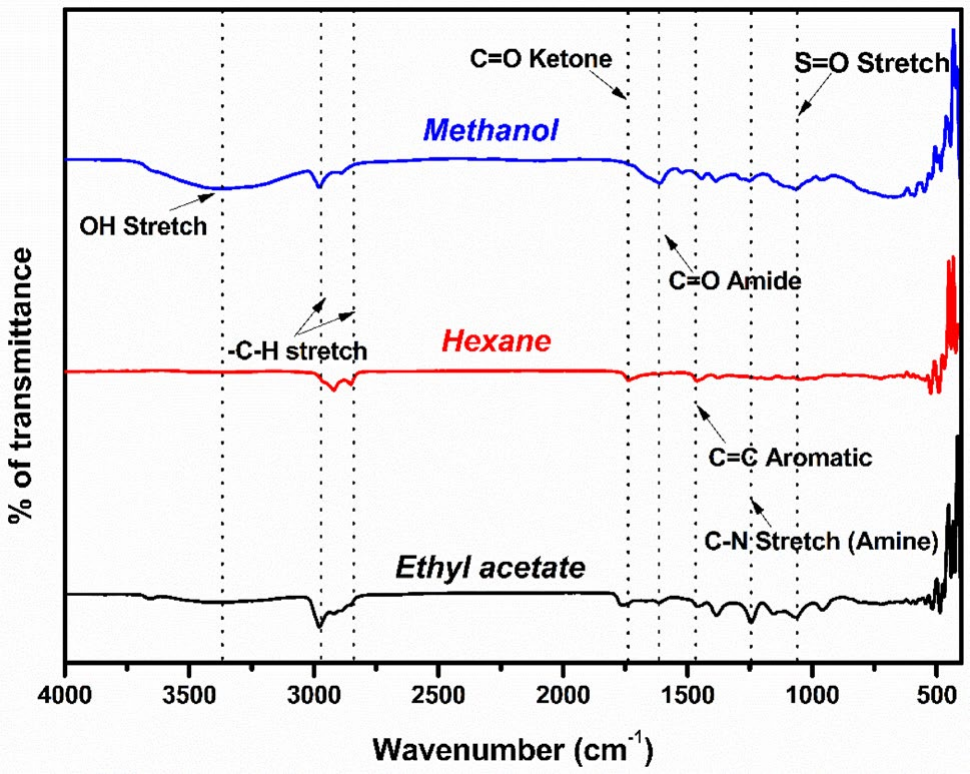
FT-IR spectrum of *A. cardiosperma* leaf extracts.

The FT-IR spectra of *A. cardiosperma* leaf extracts obtained in the 4000–500 cm^−1^ region is shown in Fig. 2. The range peaks were identical in all the extracts. Methanolic extract displayed peaks at 3390.86 cm^−1^, 2978.93 cm^−1^, 1611.66 cm^−1^, 1521.71 cm^−1^, 1443.88 cm^−1^, 1386.12 cm^−1^, 1251.20 cm^−1^ and ethyl acetate extract peaks over 3397.90 cm^−1^, 2978.48 cm^−1^, 1758.62 cm^−1^, 1615.1 cm^−1^, 1458.88 cm^−1^, 1383.05 cm^−1^, 1244.89 cm^−1^. Hexane extract showed peaks at 2919.66 cm^−1^, 2851.64 cm^−1^, 1735.4 cm^−1^, 1462.05 cm^−1^, 1379.06 cm^−1^, 1177.49 cm^−1^. Strong peaks with high-intensity bands were visible at about 3390.86 cm^−1^, 3397.90 cm^−1^ and 2919.66 cm^−1^ because of the stretching of phenols and alcohols. The peaks for the stretching bends of N–H (amines and amide), C–H (alkanes), and 2978.93 cm^−1^, 2978.48 cm^−1^, and 2851.64 cm^−1^. The peaks corresponding to alkene (C=C stretching) and ketone (C=O stretching) are 611.66 cm^−1^, 1758.62 cm^−1^, and 1735.4 cm^−1^, respectively. In peaks on 1521.71 cm^−1^, 1458.88 cm^−1^ and 1462.05 cm^−1^ indicate C≡C (aromatic) bend, 1386.12 cm^−1^, 1383.05 cm^−1^ and 1379.06 cm^−1^ represent stretching bend C–N bend (amine). The peaks over 1251.20 cm^−1^, 1244.89 cm^−1^ and 1177.49 cm^−1^ for stretching bend of S=O (sulfinyl).

### GC–MS analysis of *A. cardiosperma* leaf extracts

**Figure 3:**
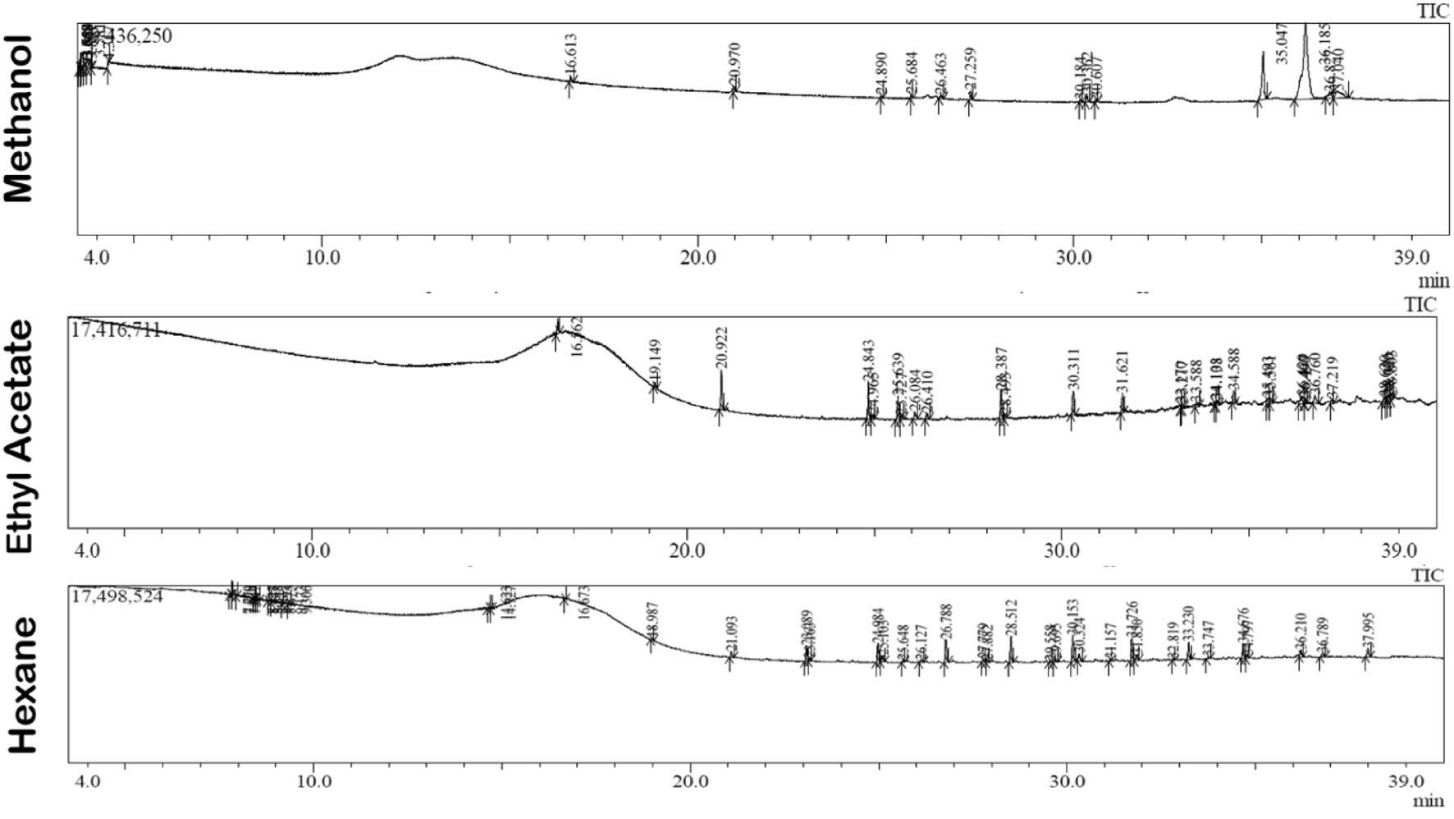
GC–MS chromatogram of the various *A. cardiosperma* leaf extract.

The chromatogram generated by the GC–MS analysis (Fig. 3 displays the percentage peaks in tandem with their retention time) of the methanolic leaf extract recognized 19/20 compounds as biologically potential (Table 2). A total of 28 compounds were found in the ethyl acetate extract, 26 of which are biologically active (Table 3). There were 40 compounds in the hexane extract; 36 are biologically active compounds (Table 4). Heneicosane (57.06%), silane, 9h-fluoren-9-ylidenebis [trimethyl (13.60%), phytol (1.51%) in the methanolic extract, 1-heptadecene (10.09%),3-hexadecene (9.99%), neophytadiene (7.66%) in ethyl acetate extract and pentadecane (9.54%), heneicosane (18.21%), octacosane, 2-methyl (14.28%) in the hexane extract. These bioactive compounds are identified based on peak area percentage and metabolic potential from *A. cardiosperma* leaf extracts.

**Table 2:** GC–MS analysis of *A. cardiosperma* leaf methanol extract.

Phyto-constituent	Retention time (min)	Area (%)
4-Bromo-*N*-[(6-methyl-2-pyridyl) aminomethyl] phthalimide	3.548	1.23
2-Propanone, 1-phenyl	3.581	1.82
1-Penten-3-yl, 5-chloro-, acetate	3.624	1.29
2,3-Dimethyloxirane	3.663	3.49
Acetaldehyde	3.750	4.20
3-Buten-2-one,4-(2,2,6-Trimethyl-7-oxabicyclo [4.1.0] Hept-1-Yl)-	3.861	0.74
Potassium trinitro methane	4.317	0.80
1-Tetradecanol	16.613	0.98
1-Pentadecene	20.970	1.06
1-Hexacosanol	24.890	0.43
Neophytadiene	25.684	0.62
Myristic acid, 9-hexadecenyl ester, (*Z*)-	26.463	0.75
Tridecanoic acid, methyl ester	27.259	1.54
8,11,14-Eicosatrienoic acid, methyl ester, (*Z*, *Z*, *Z*)	30.184	0.45
3,7,11,15-Tetramethyl-2-hexadecen-1-ol	30.362	1.51
Eicosanoic acid, methyl ester	30.607	0.54
Silane, 9H-fluoren-9-ylidenebis [Trimethyl-	35.047	13.60
Heneicosane	36.185	57.06
Azetidine, 3,3-dipentyl-	36.827	1.07
4-Etienic acid, 3-acetoxy-	37.040	6.83
Total area (%)		100

**Table 3:** GC–MS analysis of *A. cardiosperma* leaf ethyl acetate extract.

Phyto-constituent	Retention time (min)	Area (%)
9-Octadecene, (*E*)-	16.562	4.00
O-*n*-Butyl hydroxylamine	19.149	1.38
3-Hexadecene, (*Z*)-	20.922	9.99
1-Heptadecene	24.843	10.09
Nonadecane	24.965	1.30
Neophytadiene	25.639	7.66
2-Pentadecanone, 6,10,14-trimethyl-	25.727	1.94
Phthalic acid, butyl undecyl ester	26.084	2.41
3,7,11,15-Tetramethyl-2-hexadecen-1-ol	26.410	1.68
1-Nonadecene	28.387	8.40
Pentadecane	28.493	1.13
3,7,11,15-Tetramethyl-2-hexadecen-1-ol	30.311	7.47
Trifluoroacetoxy hexadecane	31.621	5.51
2-Butenamide, 2-cyano-3-hydroxy-	33.170	1.10
Sulfurous acid, dodecyl 2-propyl ester	33.217	1.29
2-Hexyldecyl acetate	33.588	2.17
*O*-isopropyl *O*-propyl* N*, *N*-Dimethyl phosphoramidate	34.103	0.82
2-Piperidinone, *N*-[4-bromo-n-butyl]-	34.138	1.91
n-Nonadecanol-1	34.588	4.31
1-Methyl-*trans*-decahydroquinol-4-ol (equat.)	35.493	2.47
Nonyl octacosyl ether	35.561	2.09
*cis*-9-Tetradecenoic acid, heptyl ester	36.420	4.83
2-(4-Fluorophenoxy)-*N*-{[4-(octyloxy)phenyl] methylidene} acetohydrazide	36.467	0.45
Oxalic acid, heptadecyl hexyl ester	36.490	1.25
Dichloroacetic acid, dodecyl ester	36.760	3.86
1-Nitrotetrahydroimidazo[4,5-*d*]imidazole-2,5(1*H*,3*H*)-dione	37.219	1.92
Cyclobutanecarboxamide, *N*-hept-2-yl-	38.720	2.50
Cyclohexanone, 2,3-dimethyl-2-(3-oxobutyl)-	38.803	2.24
Total area (%)		96.16

**Table 4:** GC–MS analysis of *A. cardiosperma* leaf hexane extract.

Phyto-constituent	Retention time (min)	Area (%)
Oxiranemethanol	7.770	0.50
Formic acid, ethenyl ester	7.828	0.28
Acetic acid, oxo-	7.947	0.22
Oxiranemethanol	8.427	0.63
Di(1,2,5-oxadiazolo) [3,4-*b*:3,4-e] pyrazine, 4,8-Diacetyl-	8.443	0.17
Glycidol	8.548	0.88
Acetaldehyde	8.793	0.51
Pyrimidine-2,4(1*H*,3*H*)-dione, 5-amino-6-nitroso-	8.875	0.37
Formic acid, ethenyl ester	9.173	0.36
Cyclobutanol	9.306	0.66
Cyclopropene	14.623	1.06
3-Butynoic acid	14.727	0.53
Argon	16.673	0.50
Tridecane, 6-methyl-	18.987	0.71
Tridecane	21.093	2.05
Tetradecane	23.089	5.58
Heptadecane,2,6,10,15-tetramethyl-	23.163	1.44
Octacosane	24.984	6.37
Pentadecane, 2,6,10,14-tetramethyl-	25.103	3.29
Neophytadiene	25.648	0.58
Bis-(3,5,5-Trimethylhexyl) phthalate	26.127	1.38
Pentadecane	26.788	7.88
1,2-Benzenedicarboxylic acid, diisononyl ester	27.779	1.32
Heptadecane, 9-octyl-	27.882	0.59
Pentadecane	28.512	9.54
2-Methyltetracosane	29.558	0.69
1-Octanol, 2-butyl-	29.695	1.55
Heneicosane	30.153	9.31
3,7,11,15-Tetramethyl-2-hexadecen-1-ol	30.324	3.30
4-Methylheneicosane	31.157	0.69
Heneicosane	31.726	8.90
1-Heptacosanol	31.850	2.49
Heptadecane, 7-methyl-	32.819	0.77
Octacosane, 2-methyl-	33.230	6.79
Di-*n*-decylsulfone	33.747	0.94
Heptadecane, 2,6,10,15 tetramethyl-	34.676	6.27
Dodecane, 4-cyclohexyl-	34.797	1.40
Octacosane, 2-methyl-	36.210	3.15
2-Ethyl-1-dodecanol	36.789	1.99
Octacosane, 2-methyl-	37.995	4.34
Total area (%)		100

### In-vitro antioxidative capacity* of A. cardiosperma* leaf extracts

#### DPPH radical scavenging assay

**Figure 4:**
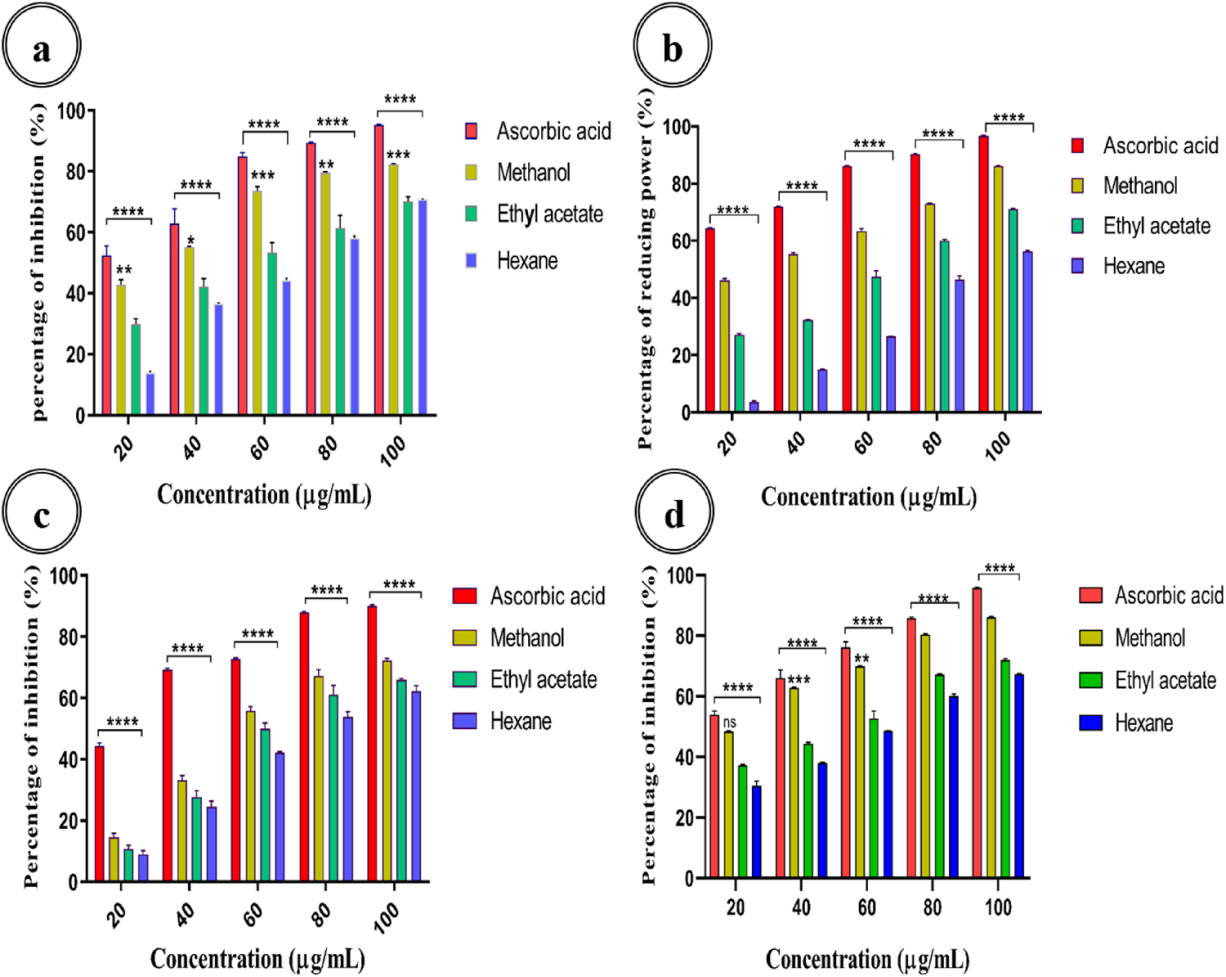
DPPH radical scavenging activity of different extracts of *A. cardiosperma* leaf (**a**). FRAP activity of different extracts of *A. cardiosperma* leaf (**b**). The metal chelating activity of various extracts of *A. cardiosperma* leaf (**c**). ABTS radical scavenging activity of different extracts of *A. cardiosperma* leaf (**d**). Data are the means ± standard error of the mean of three replicates. Different superscript asterisk indicates a significant difference between treatments, and “ns” indicates no significance (*P* < 0.05, Tukey’s multiple comparisons test).

*Aporosa cardiosperma* leaf extracts could scavenge free radicals using DPPH assay and obtained contrast outcomes with the ascorbic acid as standard (Fig. 4a). Ascorbic acid and different solvent extracts both demonstrated a notable dose-dependent reduction of DPPH radicals. However, ascorbic acid's ability to scavenge radicals outperformed and compared with these extracts. Remarkably, at 100 µg/mL, the extracts of methanol, ethyl acetate, and hexane showed increased radical-scavenging percentages of 82.35 ± 0.151%, 73.26 ± 0.361%, and 70.88 ± 0.100% respectively (Fig. 4a). The IC_50_ value for methanol extract was 28.05 µg/mL, ethyl acetate was 57.7 µg/mL, and hexane was 67.71 µg/mL. Standard ascorbic acid with d an IC_50_ value of 11.91 µg/mL.

#### FRAP assay

Based on this, the FRAP assays an electron transfer mechanism of hydrogen ions from the phenolic flavonoid compounds present in the extracts of *A. cardiosperma* leaf. The ferric dropping power of the *A. cardiosperma* extracts compared with ascorbic acid (Fig. 4b); however, reducing power by ethyl acetate (71.15 ± 0.231%) and hexane (56.26 ± 0.385%) extracts was inferior to that by methanolic extract (86.20 ± 0.117%) in 100 µg/mL concentrations.

#### Metal chelating  assay

The metal chelating activity of *A. cardiosperma* leaf extracts revealed that methanol extract showed a higher percentage of inhibition (72.32 ± 0.548%) in 100 µg/mL concentrations (Fig. 4c)compared to ethyl acetate (65.91 ± 0.429%) and hexane extracts (62.17 ± 1.84%). The antioxidant compounds have been found abundantly in the methanol extract, which increases the binding affinity to Fe ions and forms a metal complex that prevents the formation of the Fe ions-ferrozine complex.

#### ABTS radical scavenging assay

Testing the *A. cardiosperma* leaf extracts for radical scavenging activities using ABTS. Methanol extract has an elevated percentage of inhibition (86.06 ± 0.217%) compared to ethyl acetate (71.95 ± 0.42%) and hexane (67.26 ± 0.217%) in 100 µg/mL concentrations (Fig. 4d). The IC_50_ values of hexane  was 63.53 µg/mL, ethyl acetate was 44.6 µg/mL, and methanol extract was 21.25 µg/mL. The IC_50_ value of control ascorbic acid was 10.5 µg/mL. Based on the polarity of the solvents, *A. cardiosperma* leaf elucidates that the metabolite due to polar solvents has more secondary metabolites that reduce the ROS elevation. *A. cardiosperma* leaf extract's ability to quench ABTS^+^ radicals act as a hydrogen donor and could inhibit the oxidation process by shifting free radicals into stable forms.

### In-vitro antibacterial activity of *A. cardiosperma* leaf extracts

#### Agar well diffusion method

The antibacterial activity was examined in *S. aureus, B. subtilis, M. tuberculosis, A. hydrophila, K. pneumoniae, and P. aeruginosa* against different solvent extracts of *A. cardiosperma* leaf (Table 5). Compared to other solvents, methanol extract showed an increased zone of inhibition in all the tested pathogens. From the results obtained, the decrease in solvents polarity also decreased the antibacterial activity. Methanol extracts significant compounds present in the GC–MS analysis with potential antibacterial activity.

**Table 5:** Antibacterial activity of the *A. cardiosperma* leaf extracts against tested microorganisms. Each result represents the mean ± standard error of the mean (n = 3), and different superscript asterisks indicate a significant difference between the control and different concentration groups (*p* < 0.0001, Dunnett’s multiple comparisons test). “–” indicates no activity.

Zone of inhibition (mm)							
*A. cardiosperma*leaf extracts	Concentration (µg/mL)	*S. aureus*	*B. subtilis*	*M. tuberculosis*	*A. hydrophila*	*K. pneumoniae*	*P. aeruginosa*
Methanol	250	12.33 ± 0.33****	11.66 ± 0.33****	12.33 ± 0.33****	13 ± 0****	13.33 ± 0.67****	14 ± 1****
	500	14 ± 0****	13.66 ± 0.33****	14.33 ± 0.33****	14.33 ± 0.33****	15 ± 1****	16 ± 1****
	750	16 ± 0****	15 ± 0****	15.66 ± 0.33****	16 ± 0****	16.66 ± 882****	17.66 ± 0.67****
	1000	17.66 ± 0.33****	17 ± 0.577****	17.33 ± 0.33****	17 ± 0.33****	18.66 ± 882****	19.66 ± 0.33****
Ethyl acetate	250	12 ± 0****	10 ± 0****	10.66 ± 0.33****	12 ± 0****	11.66 ± 0.33****	11.33 ± 0.67****
	500	13 ± 0****	11 ± 0****	12.33 ± 0.33****	13.33 ± 0.33****	13.33 ± 0.67****	12.66 ± 0.33****
	750	14 ± 0****	12 ± 0****	13.66 ± 0.33****	14.33 ± 0.33****	15 ± 1****	13.66 ± 0.33****
	1000	16 ± 0****	13.66 ± 0.33****	14.66 ± 0.33****	15.66 ± 0.33****	16.33 ± 1.20****	15.33 ± 0.67****
Hexane	250	–	–	–	–	–	–
	500	–	–	–	–	–	–
	750	10.667 ± 0.33****	10 ± 0****	10.33 ± 0.33****	11.33 ± 0.33****	11.66 ± 0.667****	12 ± 0****
	1000	12 ± 0****	13 ± 0****	12.33 ± 0.577	12.33 ± 0.33****	12.66 ± 1****	13.33 ± 0.33****
Control (streptomycin)	25	31 ± 1	34.33 ± 0.66	26 ± 0	28.66 ± 0.33	27.33 ± 0.88	30.66 ± 1.20

#### Minimum inhibitory concentration

The MIC of the various solvent extracts from *A. cardiosperma* leaf was shown in Table 6. From the results, methanol has a potential lowest MIC value against tested microorganisms. Methanol shows minimum inhibitory concentration at 250 µg/mL concentrations in all the tested microorganisms. Additionally, ethyl acetate and hexane extracts had MICs of about 500 and 1000 µg/mL, respectively.

**Table 6:** Minimum Inhibitory Concentration (MIC) values of the *A. cardiosperma* leaf extracts against the tested microorganisms.

*A. cardiosperma* leaf extracts	Minimum inhibitory concentration (µg/mL)					
	*S. aureus*	*B. subtilis*	*M. tuberculosis*	*A. hydrophila*	*K. pneumoniae*	*P. aeruginosa*
Methanol	250	250	250	250	250	250
Ethyl acetate	500	500	500	500	500	500
Hexane	1000	1000	1000	1000	1000	1000

#### Bacterial growth kinetics

**Figure 5:**
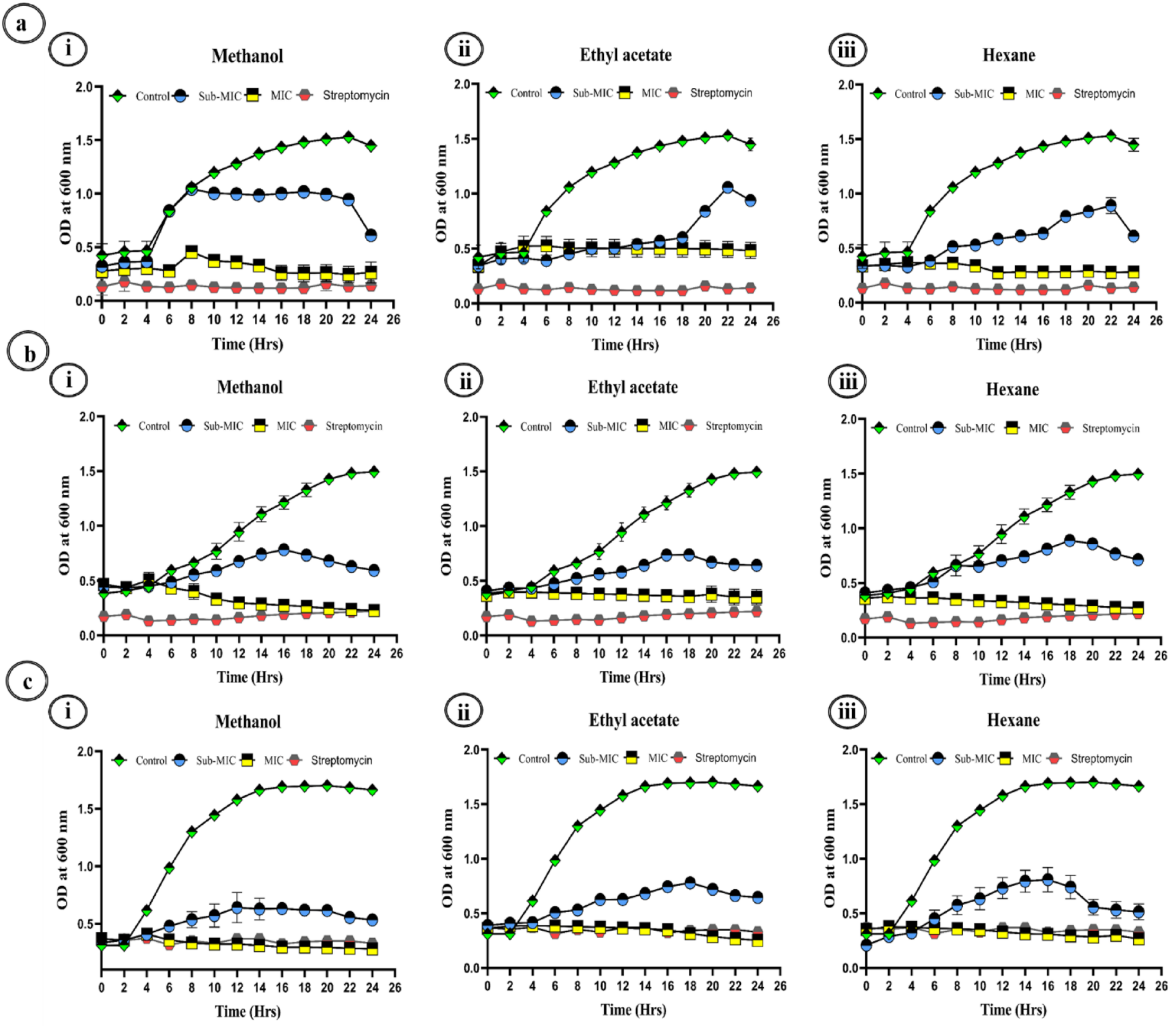
Growth curves of *S. aureus* (**a**) under the influence of various extracts of *A. cardiosperma* leaf Methanol (**i**), Ethyl acetate **(ii**), and Hexane (**iii**). Growth curves of *B. subtilis* (**b**) under the influence of various extracts of *A. cardiosperma* leaf Methanol (**i**), Ethyl acetate **(ii**), and Hexane (**iii**). Growth curves of *M. tuberculosis* (**c**) under the influence of various extracts of *A. cardiosperma* leaf Methanol (**i**), Ethyl acetate (**ii**), and Hexane (**iii**). All the experiments were compared with positive control: Streptomycin (25 µg/mL).

**Figure 6:**
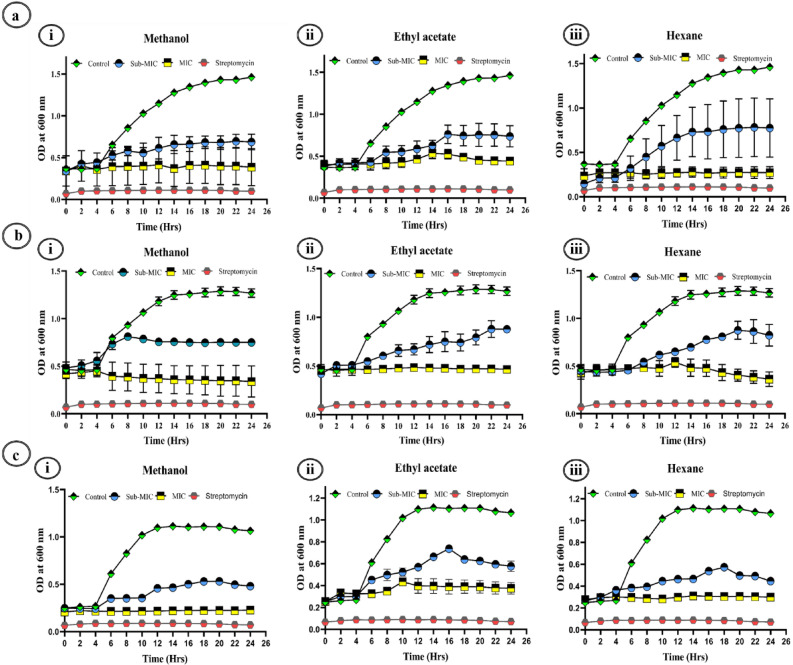
Growth curves of *A. hydrophila* (**a**) under the influence of various extracts of *A. cardiosperma* leaf Methanol (**i**), Ethyl acetate **(ii**), and Hexane (**iii**). Growth curves of *K. pneumoniae* (**b**) under the influence of various extracts of *A. cardiosperma* leaf Methanol (**i**), Ethyl acetate **(ii**), and Hexane (**iii**). Growth curves of *P. aeruginosa* (**c**) under the influence of various extracts of *A. cardiosperma* leaf Methanol (**i**), Ethyl acetate **(ii**), and Hexane (**iii**). All the experiments were compared with positive control: Streptomycin (25 µg/mL).

These findings suggest that the leaf extracts of *A. cardiosperma* and their effects on the growth of gram positive bacteria *S. aureus*, *B. subtilis*, *M. tuberculosis* were shown in Fig. 5a–c and gram negative *A. hydrophila, K. pneumoniae, and P. aeruginosa* are shown in Fig. 6a–c. The MIC and sub-MIC values of *A. cardiosperma* leaf extract were determined to examine the bacterial growth curve, along with control samples (untreated). A slight difference in growth patterns between the treated and the untreated microorganisms was observed at dosages below the MIC. However, the growth was interrupted at the MIC level. According to this investigation, various solvents in leaf extracts from *A. cardiosperma* may be able to stop the examined microbial cultures from growing at the MIC level but not at the sub-MIC level.

## Discussion

Phytochemical constituents of plant extracts decide their therapeutic activity. Because of this, methanol and water are solvents with higher quantities of phytochemicals^27^. Phenolics and flavonoids predominantly function as potent antioxidant agents and scavenge harmful free radicals, playing critical therapeutic roles in treating many inflammatory illnesses, cancer, cardiovascular diseases, and diabetes, modulating human health, and metabolism^28^. Another significant bioactive compound, tannins, has been shown to have antimicrobial, antiviral, antiparasitic, anti-ulcer, and neuroprotective properties^29^. The high quantity of polyphenolic compounds enhanced antioxidant activity since more active molecules are available to scavenge the available free radicals^30^. TPC of 107.8 μg GAE/mL for methanolic *Impatiens tinctoria* tuber extract^28^, 199.26 mg GAE/g dry weight (DW) for aqueous-methanolic pomegranate leaf extracts^31^, and 101.38 mg GAE/g DW for aqueous *Aegle marmelos* fruit extract^32^ were reported, which shows potential antioxidant and antibacterial activity. TFC of 43.50 mg QE/g DW for aqueous *Siamese senna* leaf extract^32^, 136.7 ± 0.04 μg QE/mL for methanolic *Impatiens tinctoria* tuber extract^28^, 21.53 mg/g DW for *Cassia tora* leaf extract^33^ were reported. Flavonoids are essential for metal chelation, free radical scavenging, stabilization of scavenging compounds, and inhibiting enzymes responsible for free radical production^28^. TPC and TFC, as the antioxidant activity of the *A. cardiosperma* leaf extract, were evaluated and found to be significant. TTC of 5.6 mg GAE/g for methanolic *Amaranthus lividus* leaf extract^29^ and 21 ± 1.21 mg TAE/g for ethanolic *Caulerpa racemose* extract were reported, which act as antibacterial agents.

Leaf extract from *A. cardiosperma* contains aromatic compounds, primary and secondary amines, and alkanes, which are indicators of alkaloids presence by an FT-IR analysis. *Artemisia annua* leaf has shown similar peaks in medicinal plants like stretching alcohols and phenols^34^ (3500–32,000 cm^−1^), indicating that the phenol and flavonoids could be the dominant compounds. Those functional groups carbonyl (C=O) and aromatic tertiary amine, as shown by (1371.39 cm^−1^) C–N stretch, constitute flavonoid^32^. Based on the solvent polarity, functional groups of the extract are identified^19^. Discerning distinct spectra for hexane, ethyl acetate, and methanol was possible. Each solvent contains hydroxyl and ketone groups, but only methanol and ethyl acetate possess alcoholic groups and aromatic amines. These functional groups are essential components of various secondary metabolites, including alkaloids, flavonoids, terpenoids, polyphenols, and tannins^35^.

The biologically active compounds are abundantly present in methanolic extract. Heneicosane is a significant compound present in the GC–MS analysis of methanol extracts. Heneicosane was found to exhibit excellent antimicrobial activity^36^. Silane also exhibits antimicrobial activity^37^. Ethyl acetate extract contains major compounds 1-heptadecene and 3-hexadecene. Yoon et al.^38^ reported that 1-heptadecene exhibited antifungal activity against *Plutella xylostella* larvae. Multiple therapeutic characteristics of 3-hexadecene have been reported to treat diabetes, inflammatory illnesses, and cancer^20^. Hexane extract contains major compounds like pentadecane and heneicosane. According to Bruno et al.^39^, pentadecane has an antibacterial effect on the parasites of *Leishmania infantum*. Another study found that pentadecane, a key ingredient in *Kaempferia galanga*, has strong antimicrobial properties against fungus, gram positive, and gram negative bacteria^40^.

In-vitro radical scavenging assays such as DPPH and ABTS are used to analyze the effects of *A. cardiosperma* leaf solvent extracts. The capacity of the target compounds to donate hydrogen ions or scavenge proton radicals is the crucial mechanism of both the antioxidant assays^28^. According to Durhan et al.^29^, the addition of various tannins, phenolics, and flavonoids enhances the free radical scavenging activity of *Amaranthus lividus* leaf extract, which is 75.6% of DPPH scavenging activity at 4 mg/mL concentration. 93.46% of DPPH scavenging activity at 640 µg/mL concentration of *Tetrapleura tetraptera* fruit extract reported by Okechukwu et al.^41^. Methanolic *Impatiens tinctoria* tuber extract shows 62.8% DPPH scavenging activity at 400 µg/mL concentration^28^. Methanolic *Cyperus scariosus* leaf extract shows DPPH scavenging activity with an IC_50_ value of 2.78 ± 0.21 mg/mL previously reported^42^. Also, polar solvents have more antioxidant potential than non-polar solvents, which agrees with our findings. Methanolic *Ficus carica* latex extract showed the highest ABTS radical scavenging activity, 98.81 ± 0.34%, among 18 different cultivars^43^. The FRAP assay increases the absorbance of the reaction mixture to determine the Fe^3+^–Fe^2+^ transition^44^.

2 $${\text{Fe }}{\left( {{\text{TPTZ}}} \right)_2}^{3 + }\; + {\text{ A}} - {\text{OH }} \to {\text{ Fe }}{\left( {{\text{TPTZ}}} \right)_2}^{2 + }\; + {\text{ A}}{{\text{O}}^\cdot }\; + \, {{\text{H}}^+ }$$

A-OH denotes the flavonoid and phenolic functional groups in the *A. cardiosperma* leaf extracts. Samuel et al.^45^ observed that the DPPH, ABTS and FRAP activities in the medicinal plant *Astragalus membranaceus* leaves and roots had the most significant activity, respectively. In the metal-chelating assay, an antioxidant uses an electron transfer pathway to decrease a Fe (III) salt. Copper and iron are transitional metals that increase oxidative stress. Metal ions activity can be reduced through chelation, which will minimize the production of ROS^46^. The *A. cardiosperma* methanolic extract may have a higher hydrogen-donating capacity than the ethyl acetate and hexane extracts, contributing to its higher chelating power. Aydin et al.^46^ reported *Salvia officinalis* leaf extract shows 82.8% metal chelation activity at 200 mg/mL concentration. Anand et al.^47^ also mentioned that the *Ocimum basilicum* medicinal plant methanolic extract shows a higher metal chelating capacity of 82.84% at 100 µg/mL concentration.

*A.* *cardiosperma* leaf extracts include a variety of phytochemical agents that have antibacterial properties, including steroids, diterpenes, tannins, flavonoids, saponins, and phenolic compounds. Methanol extract possesses antibacterial activity more than other solvents, when the polarity of the solvent increases, it also increases the zone of inhibition, which indicates that the extracts active secondary metabolite content is concentrated more in the polar parts^44^. Nguyen et al.^48^ found that methanolic and ethanolic extracts from the *piper beetle* Linn exhibited antibacterial effects on gram positive and gram negative microbes. According to Tambe et al.^49^, *Syzygium cumini* methanolic extract was effective against gram negative and gram positive bacteria. The gram positive bacteria (*Streptococcus pyogenes* and *S. aureus*) and gram negative bacteria (*Escherichia coli* and *P. aeruginosa*) were the four bacteria against which the methanolic extract of *Pereskia bleo* leaves showed potential antibacterial properties^50^. The hydrophilic or lipophilic character of a chemical affects its antibacterial action. Gram negative bacteria are more resistant to a considerably limited number of lipophilic substances because of their lipid-rich cell walls. Lipopolysaccharide molecules comprise its outer membrane, creating a hydrophilic environment that defends against hydrophobic chemicals^41^. This mechanism suggests that gram negative microbial species in the present study were more strongly suppressed by methanol extracts and its water component, which contains more hydrophilic compounds^51^. The antibacterial activity of a plant metabolite is mediated by disruption of microbial membranes, weakening of cellular processes, regulation of biofilm formation, inhibition of bacterial capsule synthesis, and decreased generation of microbial toxins^52^. Heneicosane was identified as the primary component present in the methanol leaf extract of *A. cardiosperma* by GC–MS analysis. According to Vanitha et al.^36^, heneicosane had antibacterial efficacy at different doses towards the bacteria *Streptococcus pneumoniae*, *M. tuberculosis*, *Bacillus cereus*, *Salmonella enteritidis*, and *Acinetobacter baumannii*. Priyadarshi et al.^53^ found that heneicos-1-ene (C_21_H_42_) had effective antibacterial action against *S. typhi* and *E. coli* at various doses. *Cardiospermum halicacabum*^54^ and *Macaranga barteri*^55^ both report neophytadiene compounds possessing antibacterial effectiveness. *Durio zibethinus* methanol extract^56^ and *Solanum macrocarpum* Linn ethyl acetate extract^57^ are the possible candidates for antibacterial action due to 1-heptadecane constituents. The presence of 3-hexadecene, in the seaweed *Caulerpa racemosa*^20^ displayed their potential antibacterial properties.

The effectiveness of an infection therapy is influenced by the choice of a therapeutic approach, which is significantly influenced by a reliable estimate of MIC^13^. *A. cardiosperma* leaf extracts with an interesting substantial MIC, excluding hexane, compared to methanol and ethyl acetate solvent extracts. These MIC results agreed with early plant extract investigations. According to Ralte et al.^35^, gram positive and negative bacteria were susceptible to different parts of *Parkia timoriana* methanolic extracts with MICs ranging from 2.19 to 7.3 mg/mL. According to Johari and Khong^50^, *Pereskia bleo* extracts were effective in four examined microorganisms: *S. aureus*, *S. pyogenes*, *P. aeruginosa*, and *E. coli*. The methanolic extract exhibited significant activity against gram positive and gram negative bacteria, with MIC value of 225 µg/mL and 450 µg/mL, respectively. Ethanolic *Calpurnia aurea* leaf crude extract showed MIC against *P. aeruginosa*, *S. pneumoniae*, and *S. aureus* was 2.5 mg/mL^27^.

The bioactive chemicals interact in the microbial cell membrane to restrict the development of microbes^19^. The subsequent interactions facilitated the active substance to permeate further the cellular cytoplasm, including the nearby protein and nucleic acids, which ultimately led to the execution of bacterial cell death^52^. Plant polyphenols are thought to have antibacterial effects, primarily via interfering with the function of bacterial cell membranes, which slows bacterial growth or reproduction^41^. In simple solutions, H_2_O_2_ exhibits notable chemical stability. Still, it swiftly undergoes a reaction with ferrous iron, generating highly reactive hydroxyl radicals (**·**OH), hydroxide anions (–OH), and oxidized ferric iron via the Fenton method reaction^58^. Numerous biomolecules, including DNA, undergo diffusion-limited reactions with the hydroxyl radical. The primary mechanism of H_2_O_2_-induced cell death is DNA damage due to hydroxyl radicals, most likely formed by contact with ferrous iron loosely associated with DNA^59^. The bioactive effect of the *Rosmarinus officinalis* and *Artemisia monosperma* leaf extracts was reported by Soliman et al.^52^ to have inhibited the development of *S. aureus*, *S. typhimurium*, *Shigella sonnei*, *Micrococcus leutus*, *Clostridium perfringens*, and *E. coli*. This was attributed to the presence of polyphenolic chemicals in the plant. Othman et al.^60^ reported inhibiting bacterial growth curves by extracts from two *Duabanga grandiflora* and *Acalypha wilkesiana* on *E. coli* and *S. aureus*. *Calpurnia aurea* leaf crude ethanol extract has suppressed the growth of *P. aeruginosa*, *S. pneumoniae*, and *S. aureus*, according to the MIC values^27^.

## Conclusion

Compared to other extracts, the methanolic extract, in particular, exhibited superior efficacy in both antioxidant assays and antibacterial activity against antibiotic-resistant pathogens. This could be the presence of the critical metabolites in the methanolic extract and the higher content of phenolic compounds, flavonoids and tannins of *A. cardiosperma* that neutralize the free radicals. These findings suggest that *A. cardiosperma* leaf extracts have substantial potential for developing herbal treatments for fish and human diseases. The phytochemical fingerprint and the potent biological activities of these extracts highlight their therapeutic potential, providing a foundation for further research into their mechanisms of action and applications in ethnobotanical medicine. This study paves the way for utilizing *A. cardiosperma* to develop natural, plant-based remedies for combating oxidative stress and bacterial infections.

## Data Availability

The data supporting this study's findings are available from the corresponding author upon reasonable request.
